# Associação entre Padrões Metabólicos Cardíacos em PET/CT Oncológico e Fatores de Risco Cardiovascular. Contribuição na Investigação Precoce de Cardiotoxicidade

**DOI:** 10.36660/abc.20260107

**Published:** 2026-04-17

**Authors:** Paola Emanuela Poggio Smanio

**Affiliations:** 1 Fleury Group São Paulo SP Brasil Fleury Group, São Paulo, SP – Brasil

**Keywords:** Oncologia, Cardiotoxicidade, Fatores de Risco, Tomografia por Emissão de Pósitrons combinada à Tomografia Computadorizada

## Introdução

O avanço das terapias oncológicas nas últimas décadas transformou radicalmente o prognóstico de diversos tipos de câncer, com isto a sobrevidas dos pacientes melhorou sobremaneira. Entretanto, esse progresso trouxe um aumento significativo de complicações cardiovasculares (CV).^1^

Nesse cenário, a cardio-oncologia se consolidou como um campo essencial, exigindo estratégias diagnósticas com acurácia cada vez maior na identificação precoce de comprometimentos cardiológicos, ainda na fase subclínica, além da implementação de planos de monitorização e controle adequados.^1^

Os agentes antineoplásicos e a radioterapia transtorácica (especialmente com doses elevadas) são responsáveis por diversos efeitos tóxicos CV que incluem disfunção ventricular esquerda (DVE), cardiomiopatias, miocardite, hipertensão arterial, doença tromboembólica, pericardite, disfunção valvar, arritmias, isquemia miocárdica, alterações vasculares, e até acometimento do ventrículo direito, que podem ser reversíveis ou permanentes.^2^

Tradicionalmente, a investigação da cardiotoxicidade (CTX) é baseada na procura de DVE pelo ecocardiograma (Eco).^1^ No entanto, este achado é observado em momento relativamente tardio no *continuum* da lesão miocárdica, sendo diagnosticado quando os danos já são irreversíveis.^2,3^

E, apesar do uso crescente de biomarcadores e do *strain* longitudinal global no Eco, ainda existe uma lacuna importante na identificação de alterações biológicas iniciais que precedam a disfunção mecânica.^2^

Com maior compreensão de potenciais CTXs associadas às novas e já estabelecidas terapias oncológicas houve avanços notáveis na disponibilidade e recursos técnicos dos exames de imagem permitindo que pacientes oncológicos possam realizar o tratamento com segurança, sem apresentar eventos ou permanecerem com sequelas cardiológicas. No entanto, a uniformidade do uso e aplicação neste contexto ainda está longe de ser ideal.

As técnicas disponíveis de medicina nuclear têm sido, há muito importantes para avaliar e monitorar pacientes em tratamento oncológico.^4^ Desde a ventriculografia radioisotópica, com alta reprodutibilidade, a cintilografia miocárdica que investiga isquemia e também a função ventricular esquerda e a cintilografia cardíaca com MIBG- ^123^I, todos muito úteis na identificação e monitoramento de CTX.^2,4,5^

A tomografia por emissão de pósitrons com 18F-Fluor-desoxi-glicoce (FDG) integrada com a tomografia computadorizada (FDG PET/CT) é amplamente utilizada para diagnóstico inicial e acompanhamento de pacientes com câncer.^1,5^

A captação de FDG reflete a utilização de glicose pelo miocárdio que apresenta notável flexibilidade metabólica, alternando entre a oxidação de ácidos graxos e o uso da glicose como principal substrato energético, de acordo com alguns fatores como a dieta que antecede o exame e algumas condições fisiopatológicas.^1^

Em condições basais, quando em jejum prolongado, o coração saudável utiliza predominantemente ácidos graxos, resultando em baixa captação miocárdica de FDG. Entretanto, a presença de estresse celular, inflamação, disfunção mitocondrial, hipóxia ou apoptose induz mudança metabólica em direção à glicólise, com aumento da captação de FDG, fato que ocorre precocemente na cascata da CTX, antes das alterações estruturais ou funcionais detectáveis pelos métodos convencionais.^6,7^

Os agentes antineoplásicos podem desencadear lesões miocárdicas por mecanismos distintos, porém convergentes nas alterações metabólicas e inflamatórias iniciais^2^

Conforme recomendação do posicionamento europeu de MN em cardio-oncologia, pacientes com maior risco de complicações CV necessitam de monitoramento precoce e contínuo padronizado para eventos agudos e para eventuais efeitos tardios.^2^ Destaca como fatores de risco (FR) para CTX: predisposição genéticas, doenças CV pré-existentes, tratamento oncológico prévio, idade, presença de FR para doença CV, o tipo da neoplasia e do tratamento oncológico realizado.^2^

Estudos previamente realizados tentaram avaliar o uso do FDG PET/CT na detecção precoce de CTX, demonstrando, inclusive, verificando a associação entre os padrões de captação cardíaca de FDG e os parâmetros metabólicos na identificação de CTX.^6–8^

Entretanto, estudo prospectivo multicêntrico mais recente avaliando ^18^FDG PET/CT, *strain* miocárdico e biomarcadores durante a quimioterapia em portadores de linfoma, observou que o aumento da captação de FDG foi achado frequentemente observado, mas sem correlação com presença de disfunção ventricular esquerda (DVE). Desta forma, a relevância clínica da regulação metabólica miocárdica ainda requer investigação adicional.^9^

Também permanece ainda incerta a associação entre os padrões de captação miocárdica de FDG e os FR CV tradicionais em pacientes oncológicos.

Ribeiro Sobrinho et al.^10^ analisaram, de forma pioneira, em grande coorte, a associação entre padrões de captação miocárdica de FDG em exames oncológicos de PET/CT e FR CV, demonstrando que sexo masculino e maior peso corporal são preditores independentes de captação miocárdica, enquanto diabetes mellitus e doença arterial coronariana se associam à sua ausência. Esses achados podem reposicionar a captação de FDG como um marcador metabólico CV relevante, e não apenas como um achado incidental.

**Figura 1:**
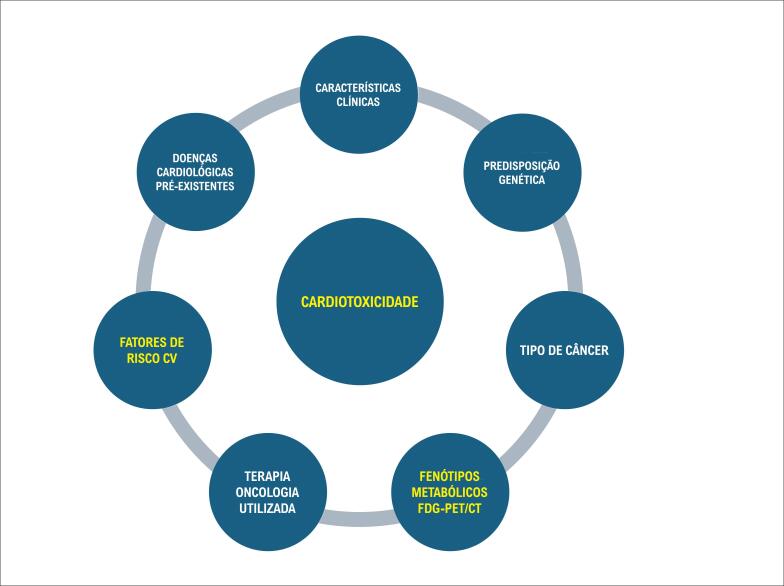
Fatores de risco para o desenvolvimento de cardiotoxicidade e fenótipos metabólicos no FDG PET/CT. CV: cardiovascular.

Mas, ainda se faz fundamental interpretar o aumento da captação de FDG de forma contextualizada, integrando características clínicas, metabólicas e CV dos pacientes. A simples dicotomia entre presença ou ausência de captação pode ser entendida como a porta de entrada para uma classificação mais ampla: desde um fenótipo metabolicamente neutro, passando por um remodelamento adaptativo potencialmente reversível, até estágios inflamatórios e metabolicamente descompensados, associados a maior risco de DVE futura (Figura 1).

Em um cenário de cardio-oncologia integrada, FDG PETCT realizado rotineiramente para pacientes oncológicos pode fornecer informações adicionais valiosas sobre o estado metabólico do coração, sem aumento de custo ou exposição adicional à radiação.^5^

Algumas limitações devem ser consideradas, sugerindo a necessidade de estudos prospectivos futuros. O desenho retrospectivo e transversal impede inferências prognósticas, e a avaliação visual da captação, embora pragmática e alinhada à prática clínica, está sujeita à variabilidade interobservador e à influência do preparo metabólico. Ainda assim, o grande tamanho amostral, a análise multivariada e a coerência fisiopatológica dos achados conferem robustez às conclusões.

**Figura 2:**
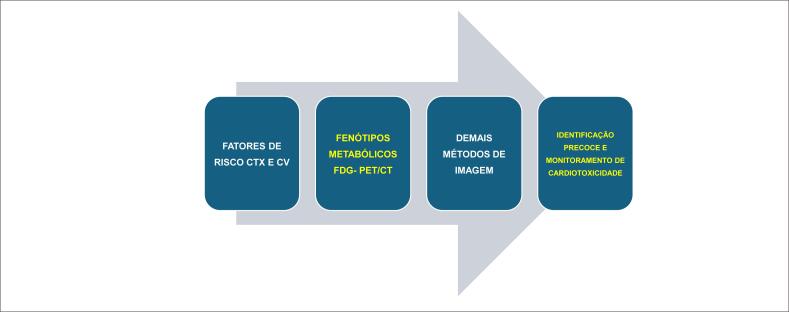
Estratégia sugerida para identificação precoce, prevenção e monitoramento de cardiotoxicidade.

O principal legado deste estudo é reforçar a necessidade de abandonar uma interpretação simplista da captação miocárdica de FDG. A imagem metabólica deve ser entendida como parte de um *continuum* biológico, integrando as características clínicas, os FR dos pacientes, a terapia utilizada e a vulnerabilidade miocárdica (Figura 2).

Em síntese, o artigo publicado nesta edição dos Arquivos Brasileiros de Cardiologia amplia o horizonte da cardio-oncologia ao demonstrar que a captação miocárdica de FDG no PET/CT reflete fenótipos CV distintos, modulados por FR tradicionais. Trata-se de um passo relevante em direção a uma abordagem metabólica da CTX associada ao tratamento oncológico.
