# Supplementary material for: Associação entre Padrões Metabólicos Cardíacos em PET/CT Oncológico e Fatores de Risco Cardiovascular. Contribuição na Investigação Precoce de Cardiotoxicidade
Source: Arq Bras Cardiol. 2026 Apr 17;123(1):e20260107. [Article in Portuguese] doi: 10.36660/abc.20260107 (PMC13127809; doi:10.36660/abc.20260107)
Supplement: Translation

# Translation: Association between Cardiac Metabolic Patterns in Oncological PET/CT and Cardiovascular Risk Factors. Contribution to the Early Investigation of Cardiotoxicity

**DOI:** 10.36660/abc.20260107i

**Authors:** Paola Emanuela Poggio Smanio

**Affiliations:** 1 São Paulo SP Brazil Fleury Group, São Paulo, SP – Brazil

**Keywords:** Medical Oncology, Cardiotoxicity, Risk Factors, Positron Emission Tomography Computed Tomography

## Introduction

The advancement of cancer therapies in recent decades has radically transformed the prognosis of various types of cancer, significantly improving patient survival. However, this progress has brought a significant increase in cardiovascular (CV) complications.^1^

In this scenario, cardio-oncology has established itself as an essential field, requiring diagnostic strategies with ever-increasing accuracy in the early identification of cardiological impairments, even in the subclinical phase, in addition to the implementation of appropriate monitoring and control plans.^1^

Antineoplastic agents and transthoracic radiotherapy (especially at high doses) are responsible for several toxic CV effects, including left ventricular dysfunction (LVD), cardiomyopathies, myocarditis, hypertension, thromboembolic disease, pericarditis, valvular dysfunction, arrhythmias, myocardial ischemia, vascular changes, and even right ventricular involvement, which may be reversible or permanent.^2^

Traditionally, cardiotoxicity (CTX) investigation is based on the search for left ventricular dysfunction (LVD) by echocardiography (Echo).^1^ However, this finding is observed relatively late in the *continuum* of myocardial injury, being diagnosed when the damage is already irreversible.^2,3^

And, despite the increasing use of biomarkers and global longitudinal *strain* in Echo, there is still a significant gap in the identification of early biological changes that precede mechanical dysfunction.^2^

With a greater understanding of potential CTXs associated with new and established cancer therapies, there have been remarkable advances in the availability and technical resources of imaging exams, allowing cancer patients to undergo treatment safely, without experiencing events or remaining with cardiologic sequelae. However, the uniformity of use and application in this context is still far from ideal.

Available nuclear medicine (NM) techniques have long been important for evaluating and monitoring patients undergoing cancer treatment.^4^ From radioisotopic ventriculography, with high reproducibility, to myocardial scintigraphy, which investigates ischemia and also left ventricular function, and cardiac scintigraphy with MIBG-^123^I, all are very useful in the identification and monitoring of CTX.^2,4,5^

Positron emission tomography with 18F-Fluorodeoxyglucose (FDG) integrated with computed tomography (FDG PET/CT) is widely used for initial diagnosis and follow-up of cancer patients.^1,5^

FDG uptake reflects the use of glucose by the myocardium, which exhibits remarkable metabolic flexibility, alternating between fatty acid oxidation and the use of glucose as the main energy substrate, according to factors such as the diet preceding the study and some pathophysiological conditions.^1^

Under basal conditions, during prolonged fasting, a healthy heart predominantly uses fatty acids, resulting in low myocardial FDG uptake. However, the presence of cellular stress, inflammation, mitochondrial dysfunction, hypoxia, or apoptosis induces a metabolic shift toward glycolysis, with increased FDG uptake, which occurs early in the CTX cascade, before structural or functional changes detectable by conventional methods.^6,7^

Antineoplastic agents can trigger myocardial lesions through distinct mechanisms, but are convergent in the initial metabolic and inflammatory changes.^2^

As recommended by the European MN position statement in cardio-oncology, patients at higher risk of CV complications require early and continuous standardized monitoring for acute events and possible late effects.^2^ It highlights the following as risk factors (RF) for CTX: genetic predisposition, pre-existing CV diseases, previous cancer treatment, age, presence of RF for CV disease, the type of cancerand cancer treatment performed.^2^

Some previously conducted studies have attempted to evaluate the use of FDG PET/CT in the early detection of CTX, demonstrating, among other things, the association between cardiac FDG uptake patterns and metabolic parameters in the identification of CTX.^6–8^

However, a more recent prospective multicenter study evaluating ^18^FDG PET/CT, myocardial *strain*, and biomarkers during chemotherapy in lymphoma patients observed that increased FDG uptake was a frequently observed finding, but without correlation with the presence of left ventricular dysfunction (LVD). Thus, the clinical relevance of myocardial metabolic regulation still requires further investigation.^9^

The association between myocardial FDG uptake patterns and traditional CVRFs in cancer patients also remains uncertain.

Ribeiro Sobrinho et al.^10^ pioneered a large cohort study analyzing the association between myocardial FDG uptake patterns in oncological PET/CT scans and CV FR, demonstrating that male sex and higher body weight are independent predictors of myocardial uptake, while diabetes mellitus and coronary artery disease are associated with its absence. These findings may reposition FDG uptake as a relevant CV metabolic marker, and not just as an incidental finding.

However, it is still essential to interpret the increase in FDG uptake in a contextualized way, integrating the clinical, metabolic, and CV characteristics of patients. The simple dichotomy between the presence or absence of uptake can be understood as the gateway to a broader classification: from a metabolically neutral phenotype, through a potentially reversible adaptive remodeling, to inflammatory and metabolically decompensated stages, associated with a higher risk of future DVE (Figure 1).

**Figure 1:** Risk factors for the development of CTX and metabolic phenotypes in FDG PET/CT.

In an integrated cardio-oncology setting, routinely performed FDG PETCT for cancer patients can provide valuable additional information about the metabolic state of the heart, without increased cost or additional radiation exposure.^5^

Some limitations should be considered, suggesting the need for future prospective studies. The retrospective and cross-sectional design prevents prognostic inferences, and the visual assessment of uptake, although pragmatic and aligned with clinical practice, is subject to interobserver variability and the influence of metabolic preparation. Still, the large sample size, multivariate analysis, and pathophysiological consistency of the findings lend robustness to the conclusions.

The main legacy of this study is to reinforce the need to abandon a simplistic interpretation of myocardial FDG uptake. Metabolic imaging should be understood as part of a biological *continuum*, integrating clinical characteristics, patient RF, the therapy used, and myocardial vulnerability (Figure 2)..

**Figure 2:** Suggested strategy for early identification, prevention, and monitoring of CTX.

In summary, the article published in this issue of the *Arquivos Brasileiros de Cardiologia* broadens the horizon of cardio-oncology by demonstrating that myocardial FDG uptake on PET/CT reflects distinct CV phenotypes, modulated by traditional RF. This is a significant step towards a metabolic approach to CTX associated with cancer treatment.
